# “Nihilism” of chronic heart failure therapy in children and why effective therapy is withheld

**DOI:** 10.1007/s00431-016-2700-3

**Published:** 2016-02-19

**Authors:** Dietmar Schranz, Norbert F. Voelkel

**Affiliations:** Pediatric Heart Center, Justus-Liebig University Clinic, Feulgenstr. 12, 30385 Giessen, Germany; School of Pharmacy, Virginia Commonwealth University, Richmond, VA USA

**Keywords:** Chronic heart failure, Infants and children, Bisoprolol, Lisinopril, Spironolactone, Pulmonary artery banding

## Abstract

Major advances in chronic heart failure (cHF) therapy have been achieved and documented in adult patients, while research regarding the mechanisms and therapy of cHF in children has lagged behind. Based on receptor physiological studies and pharmacological knowledge, treatment with specific ß1-adrenergic receptor blocker (ARB), tissue angiotensin-converting enzyme inhibitor (ACE-I), and mineralocorticoid antagonists have to be recommended in children despite lack of sufficient data derived from prospective randomized studies. At our institution, bisoprolol, lisinopril, and spironolactone have been firmly established to treat systolic cHF, hypoplastic left heart syndrome (HLHS) following hybrid approach and congenital left-right shunt diseases, latest in patients where surgery has to be delayed. Chronic therapy with long-acting diuretics and fluid restriction are not advocated because short-term effects are achieved at the expense of further neuro-humoral stimulation. It remains unclear why diuretics are recommended although evidence-based studies, documenting long-term benefit, are missing. However, that is true for all currently used drugs for pediatric cHF.

*Conclusion*: This review focuses on the prevailing “nihilism” of cHF therapy in children with the goal to encourage physicians to treat pediatric cHF with a rationally designed therapy, which combines available agents that have been shown to improve survival in adult patients with cHF. Because of the lack of clinical trials, which generate the needed evidence, surrogate variables like heart and respiratory rate, weight gain, image-derived data, and biomarkers should be monitored and used instead. The recommended pharmacological therapy for systolic heart failure is also provided as the basis for utilizing reversible pulmonary arterial banding (PAB) as a novel strategy in young children with dilative cardiomyopathy (DCM) with preserved right ventricular function.

**Table:** 

**What is Known:** • *Heart failure (HF) in children is a serious public health concern.* • *HF has numerous etiologies, but unspecific symptoms.* • *HF interplays among neuro-humoral, and molecular abnormalities.* • *Pediatric cHF-therapy is currently based on loop-diuretics, fluid restriction and digoxin.*
**What is New:** • *Cardiac function analysis has to include cardiac synchrony and VVI.* • *Considering enormous potential of cardiac regeneration, therapy has to extend with selective ß1-ARB, tissue ACE-I and mineralocorticoid blockers, loop-diuretics avoided as ever possible.* • *Inhibition of the endogenous neuro-humoral stimulation is monitored by surrogate parameters as heart and breath rate and systolic and diastolic blood pressure.* • *Advocated HF therapy serves for regenerative strategies as reversible Pulmonary Artery Banding in DCM.*

**Electronic supplementary material:**

The online version of this article (doi:10.1007/s00431-016-2700-3) contains supplementary material, which is available to authorized users.

## Considerations

### “Science is neither dogmatic nor democratic!”

Heart failure is the final stage of a wide variety of cardiac diseases. Symptoms of HF develop, once the heart becomes unable to meet the metabolic demands of the body.

In adults, heart failure is frequently called the new epidemic of the twenty-first century [36]. Causes of heart failure in childhood are associated to congenital heart diseases, cardiomyopathies, and arrhythmias as well as acquired heart and circulatory diseases [21]. Epidemiological studies covering the pediatric age group are very rare. More than 20 years ago, Rodeheffer et al. [46] reported an incidence of four infants <1 year of age in 1000 person years and a prevalence in children <10 years of age of 1.3 in 1000.

Hsu and Pearson summarized the various causes of pediatric heart failure [21] as well as the current treatment and future directions [22].

**Fig. 1:**
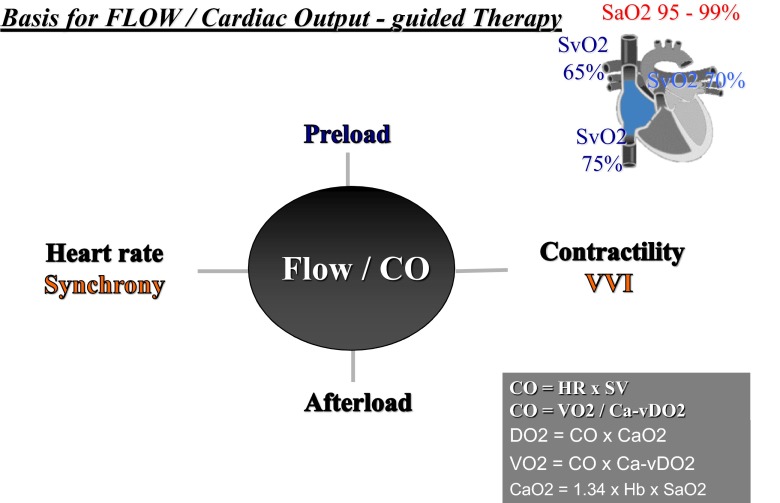
Parameters responsible for cardiac output. In addition to heart rate, contractility, preload, and afterload, cardiac synchrony as well as ventricular-ventricular interaction (VVI) defines cardiac function. *CaO2* oxygen content, *Ca-vDO2* arterial-venous oxygen content difference, *CO* cardiac output, *DO2* oxygen delivery, *HR* heart rate, *SaO2* arterial oxygen saturation, *SvO2* venous oxygen saturation in superior and inferior caval vein, *SV* stroke volume, *VO2* oxygen consumption

Considering that cardiac output (flow) is based on heart rate, myocardial contractility, preload, afterload, synchrony, and ventricular-ventricular interaction (VVI), a low cardiac output might be associated with heart failure due to one or all of these variables (Fig. 1).

If there is no successful strategy to remove the root cause, the aims of chronic congestive heart failure (CHF) therapy are to modify the neuroendocrine responses that worsen CHF and their pathophysiological consequences and to stimulate endogenous repair mechanisms.

Established therapies in adults aim at reducing preload, afterload, and neuro-humoral activation and to halt the ongoing loss of cardiomyocytes, which gives way to replacement fibrosis [7]. In Table 1, therapeutic goals for chronic HF treatment in children are summarized.

**Table 1:** Therapeutic goals for chronic HF treatment in children

1. Preload optimization by avoiding intravascular, in particular intra-arterial, volume depletion
2. Reduction of the systemic vascular resistance without jeopardizing the coronary perfusion pressure
3. Optimizing myocardial oxygen consumption and re-establishing myocardial synchrony as well as VVI
4. Allowing time to establish endogenous and exogenous repair mechanisms

In this context, tachy- and brady-arrhythmias need to be prevented and if present effectively treated; sinus rhythm heart rate needs to be adjusted to the lowest effective level in order to reduce myocardial oxygen demand and to optimize the diastolic ventricular filling time. Chronic stimulation of a dysfunctional myocardium is counterproductive; all exogenous therapeutic strategies that stimulate the neuro-humoral system have been repeatedly analyzed and ought to be, whenever possible, omitted (for example: chronic treatment with loop-diuretics); if inotropic agents and vasoconstrictors become necessary, they should be used as short as possible or as a bridge towards heart transplant, if cardiac assist devices are not the better option.

Considering the balance of oxygen delivery and consumption, positive inotrope vasodilators (milrinone, levosimendane) ought to be preferentially used as long as myocardial perfusion pressures are not compromised. During catecholamine infusion therapy, strategies designed to protect the myocardium should be considered, for example: a combination of epinephrine or norepinephrine infusion with ß1-receptor blockers. In decompensated systolic heart failure, which often occurs in infants and children with dilated cardiomyopathy, short-term epinephrine infusion combined with intravenous or oral ß1-receptor blocking agents (metoprolol, bisoprolol) is not a contradiction, but an actually used and recommended strategy [43]. In addition, it has to emphasize that the right and left heart does not act in isolation; cardiac re-synchronization [35] as well as strategies to improve the ventricular-ventricular interaction (VVI) is desirable in order to allow endogenous cardiac repair, in particular in younger patients [53, 61].

Bridging to heart transplantation (HTX), with or without the use of assist devices, or transiting the patient from an acute to a chronic heart failure status might be the therapeutically strategy of choice, if no satisfactory return to normal function can be achieved [53].

## Differences of chronic HF treatment in children and adults

Age- and perhaps mechanism-independent chronic HF is associated with neuro-humoral activation and increased levels of circulating neuro-hormones (noradrenaline, adrenaline, renin, angiotensin II, aldosterone, vasopressin), which lead to vasoconstriction, sodium, and water retention. Continuous endogenous neuro-humoral activation leads to myocardiocyte apoptosis, necrosis, and cardiac fibrosis, the main causes of chamber dilatation and progressive dysfunction, culminating in a vicious cycle of ever worse quality of myocardium and heart function [51].

Medical treatment recommendations for chronic HF in adults have been based on controlled, randomized studies [29]. Large cohort studies were necessary to pinpoint that among a group of HF treatment drugs as ß-adrenergic receptor blockers [2, 3, 40], angiotensin-converting enzyme (ACE) inhibitors [1] and aldosterone receptor antagonists [41] significantly reduce the mortality by counteracting the neuro-humoral overdrive of chronic HF. Such large cohort clinical trials cannot realistically be conducted in children with chronic heart failure due to the small patient numbers and the heterogeneity of the HF causes. Clinical trials in the pediatric age group are generally underpowered and cannot detect significant impact differences on survival rates. Several review articles address the differences in responses to medications in relation to the age of patients, pharmacokinetic/pharmacodynamic characteristics, and underlying causes of CHF and their molecular characteristics [48, 51]; however, the overall strategy to block the neuro-humoral axis is not disputed. Rossano and Shaddy [48] pointed to the missing data in children and emphasized that extrapolating evidence from adult patients to children with heart failure may have limited utility. However, given the state of our current situation of pediatric heart failure therapy, this statement lacks a forward-looking attitude and encourages a not-justified therapeutic “nihilism” when it comes to chronic HF treatment in children. Unfortunately, this entirely uncreative state of the affairs has been cultivated over decades [49].

In adults with chronic HF, new therapeutic strategies like angiotensin-neprilysin inhibition have been lauded as a paradigm shift in HF therapy [30], while in children, the use of ACE inhibitors, ß-adrenergic receptor blockers, and mineralocorticoid-receptor blockers still remains controversial and—if recommended—is rarely used [60]. This then begs the question, why effective therapy is being withheld in infants and children despite well-designed and sufficiently powered randomized trials in adults, which have been published more than a decade ago? In fact, drugs shown to be highly effective in adult heart failure patients have been discredited because of their use in studies with an unfortunate design, despite or because of their administration in controlled, randomized, double-blind trials which are acknowledged as the gold standard in evidence-based medicine. In one single controlled randomized study, the ACE inhibitor enalapril was judged to be not effective for the treatment of univentricular heart failure [23]; this study has been frequently cited to support the notion that ACE inhibitors are ill-advised in the treatment of all children with HF. ß-Adrenergic receptor (BAR) blockers have also been labeled to be of no benefit for children with chronic HF; the potential use of highly selective or non-specific BAR blocker has not been considered. In one, highly cited BAR-blocker study in children, the effect of carvedilol was assessed in 106 patients, while placebo was administered to 54 children with functional classes II and III [56]. This study, performed in 26 North American pediatric heart centers, was not stratified according to the causes and severity of HF and terminated after 5 years; in addition, there was no control for co-medications; the dosage of diuretic drugs and whether the BAR-blocker dose was adequate had not been or monitored by simply observing the heart rate response. Remarkably, during the same time period in US pediatric heart centers, BAR blockers were routinely used only in 4 % of children with cHF [60]. This fact illustrates what little clinical experience there is with BAR-blocker therapy in children with cHF.

The third thrust of HF therapy should decrease the endogenous neuro-hormonal response by mineralocorticoid antagonism. In the RALES trial [41], treatment with spironolactone, in addition to conventional therapy, led to a relative reduction of the risk of death by 30 % and of cHF-related hospitalizations by 35 % in adults. In pediatric patients, spironolactone is a common component of diuretic regimens due to its potassium-sparing property but seldom used as a tissue aldosterone antagonist to influence cardiac re-remodeling, in particular myocardial fibrosis. The Canadian guideline reports that therapy with drugs that block the effects of aldosterone is well established in adults with systolic HF; “data regarding the role of spironolactone or related agents in the treatment of children with cHF are very limited” [25]. However, the few pediatric data published by Masutani et al.—with a focus on the importance of aldosterone-blocking drugs like spironolactone in children with HF, and in particular with preserved ejection fraction (HFpEF), have been widely ignored [28]. On the other hand, since 1978, Engle et al. [16] had administered 137 courses of furosemide to 106 hospitalized pediatric patients with salt and water retention associated with cardiac or renal disease; furosemide is by far the most commonly used and recommended drug, not only for acute but also for chronic heart failure in children. The diuretics are recommended as effective and safe in the pediatric age group when administered acutely as a parenteral medication and over a long-term course by the oral route in the doses and at the time intervals used in this study [16]. Yet, despite a lack of evidence-based studies, the use of diuretics is supported by Cochrane’s systematic review [18] as sufficient for the routine use in children with chronic CHF. While there is a recognized lack of evidence with regard to the use of ACE inhibitors, BAR blockers, and mineralocorticoid antagonists in pediatric patients, the chronic use of diuretics has never been scrutinized by pediatric opinion leaders [25]. A similar situation prevails with regard to digoxin treatment of chronic heart failure in infants and children. Again, a randomized, placebo controlled study is only available for adult patients with ischemic or cardiomyopathic CHF. Digoxin had shown weak, but positive effects on left ventricular ejection fraction (EF), exercise capacity, quality of life, and reduction of hospitalizations, but the survival rate in the whole cohort was not significantly improved [14]. In the pediatric literature, we find several early clinical observational studies, which demonstrated a beneficial effect on CHF, including improved contractility and a decreased neuro-hormonal stimulation. Perhaps the Na/K-ATPase inhibitor digoxin, as a positive inotropic drug might be indicated in younger patients. On the other hand, a recently published study of 48 infants with chronic HF secondary to left-to-right shunt lesions who were randomized to treatment with enalapril and furosemide ±, digoxin did not find any clinical improvement. It was concluded that digoxin does not provide any extra benefit in the treatment of such patients [15]. In considering the results of this latter study, the Canadian guidelines for pediatric chronic HF did not recommend digoxin in children with chronic HF [25]. However, digoxin is a weak vasoconstrictor and co-therapy with a neuro-hormonal axis-stimulating diuretic is from our point of view not indicated for HF due to a left-right shunt because the shunt flow is favored.

In conclusion: HF drugs need a clearly defined indication and a convincing rationale. Simply, a randomized, placebo controlled study design—which is highly desirable—however, lacking a crisp definition of the clinical target and the underlying disease mechanism does not allow us to generate the needed outcome information. One wonders also why the effectiveness of a chronic HF drug is not reported, as it is customary for the drugs targeting pulmonary arterial hypertension [29]; i.e., if the drug does not significantly alter the mortality, then surrogate endpoints which demonstrate improvement of the clinical status should be obtained to support their use. PH-targeting drugs are not being withheld if they do not affect mortality but improve the clinical functional class [26, 61].

## Recommendations to improve chronic HF treatment in infants and children

### Systolic dysfunction related to left ventricular dilated cardiomyopathy

**Fig. 2:**
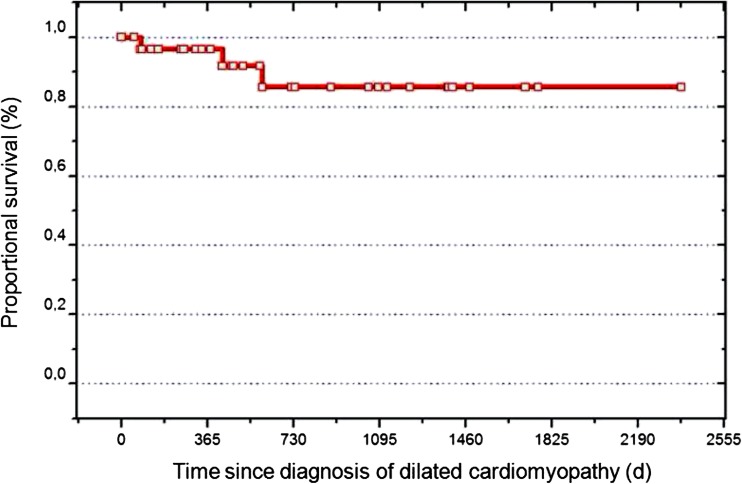
Estimated survival and freedom of death in children with DCM [50]. Shown is the median follow-up 16 months (range 2–80 months) of 38 children in an age less than 3 years admitted at the Pediatric Heart Center Giessen. The Kaplan-Meier survival curve after the diagnosis of dilated cardiomyopathy revealed a 1-year survival of 97 % and a 5-year survival of 86 % [50]

The incidence of childhood cardiomyopathy is in the range of 1.13–1.24 per 100,000 children according to the data collected by two population-based registries, where dilated cardiomyopathy accounts for more than half of the primary causes [6, 27]. Dilated cardiomyopathy (DCM) is a serious disease. The 1-year and 5-year rates of death or transplantation were first described to be 35 and 49 %, respectively, in a population-based study in 1998 and 31 and 46 % in the largest population study in 2006 [39, 56]. Recently, we described in a single-center study a lower rate for death of 18 % and transplantation 31 % [50], which is perhaps the result of an improved treatment strategy (Fig. 2). While the overall goal of the treatment of children with DCM is to avoid death or delay heart transplantation, different survival rates after the diagnosis of DCM may be attributed to different treatment strategies. Prior to treatment, a detailed diagnostic workup is needed for left ventricular (LV)-DCM: this includes the medical history, clinical examination, echocardiogram, X-ray, MRI, coronary-angiography, and myocardial biopsy, as well as laboratory data (BNP or NT-proBNP, CRP, hemoglobin, sodium, potassium, creatinin, albumin, and if possible, plasma levels of aldosterone, norepinephrine, angiotensin, renin, respectively). Treatment success is monitored by the clinical functional (NYHA, Ross I–IV) and nutritional status, heart and respiratory rate, intermittent diastolic and systolic blood pressure, and SaO_2_ measurements. Follow-up laboratory and imaging data should be acquired.

Taking into account-specific exclusion criteria, at our institution, the chronic, age-independent heart failure therapy consists of specific long-acting ß1-adrenoreceptor blocker (bisoprolol), long-acting tissue angiotensin-converting enzyme inhibitor (lisinopril), and mineralocorticoid-receptor blocker (spironolactone) and applying a goal-oriented drug dosage [43]. Digoxin is the fourth-line HF drug with a target of a plasma level of 0.5–0.9 ng/ml. Chronic treatments with loop-diuretics are avoided, and hydrochloro-thiazide in low dosages of 0.5–1 mg/kg are applied once or twice per day, if really needed. If there are signs of inappropriate ADH secretion with severe hyponatremia, we currently use the V2-receptor antagonist, tolvaptan, once per day in a dosage of 0.1–(0.3) mg/kg, targeting a sodium serum level of 140–145 mmol/l. The hypothesized treatment goals for treatment of LV-DCM with a highly specific ß1-BAR blocker, tissue ACE inhibitor, and aldosterone-antagonist are summarized in Table 2.

**Table 2:** Summarizes the hypothetical aim of therapy and the used heart failure drugs

1. *Heart rate control* in order to improve the ratio of (myocardial) oxygen consumption to demand and prolong the time for diastolic ventricular filling (ß1-specific ß-blocker)
2. *Diminishing* apoptosis and myocytes necrosis (ß1-specific ß-blocker)
3. *Diminishing* interstitial fibrosis by blocking sympathetic- and RAA-S (three first-line drugs)
4. *Reduction* of cardiac afterload together with *preservation* of the coronary perfusion pressure by adequate (preload) intravascular, in particular, arterial vascular filling (avoidance of diuretics, effectively dosed ACE-I + ß1-specific blocker, preserving the beta 2 receptor function)
5. *Basis for additional strategies* to re-establish ventricular synchrony and re-establish VVI as prerequisite for cardiac regeneration (all three first-line drugs)
6. *Low risk-benefit ratio* and *high parental compliance* by daily single-dose therapy together with easy dosing of 0.1–0.2 (0.3) mg/kg × day for both bisoprolol and lisinopril and once application of spironolactone 1–2 mg/kg × day, respectively

Considering the (patho-)physiology of the ß-adrenergic receptors (ß-AR) in pediatric DCM, highly selective ß1-AR blockers are recommended in children to treat chronic HF caused by LV-DCM [34, 43, 50]. It is generally accepted that chronic stimulation of the cardiac β1-adrenergic system is toxic to the heart and contributes to the pathogenesis of congestive heart failure [11]. We could show that children that underwent open heart surgery with cardiac arrest demonstrated a decreased ß-adrenoceptor-mediated adenylate cyclase activation in a manner compatible with an uncoupling of ß-adrenoceptors from the Gs-protein-adenylate cyclase complex [52]. Cardiac arrest and HF liberate myocardially stored norepinephrine [47] and cause ß1-receptor desensitization. As stated, chronic stimulation of β1-receptors is cardiotoxic while β2-receptor stimulation might be cardio-protective [8, 11]. The elegant study of Miyamoto et al. [33, 34] not only demonstrated the differences of BAR pathophysiology between pediatric and adult patients with idiopathic dilated cardiomyopathy but also recommended the use of ß1-selective BAR blocker in children. The data by Miyamoto et al. might also explain the failure of non-specific ß-blocking therapy in previous clinical pediatric HF trials. Miyamoto et al. showed that ß1-ARs are downregulated in both adults and children with chronic HF but that the ß2-AR is downregulated only in pediatric DCM. She stated “Further inhibition of the already downregulated ß2-ARs may override the benefit of ß1-AR inhibitor therapy, because preservation of ß2-AR function is beneficial.” In addition, several studies support the cardio-protective effect of short-term beta-2 stimulation [4, 5, 9, 17, 31, 32, 38, 45, 63]. Bisoprolol is a long-acting, highly cardio-selective BAR-blocker, with a low side effect profile. Bisoprolol has a beta1/beta2-specific binding ratio of about 125, metoprolol of about 80, and propranolol and carvedilol of about 5 [63]. Dual mechanism of action includes selective beta1-receptor blockade and stimulation of endothelial NO production [62]. Additionally, ß1-specific BAR-blockers can block renal ß1-adrenergic receptors and concomitant renin release caused by HF or in association with ACE inhibitor therapy [10].

Combined with ß1-adrenoreceptor blockers, long-acting tissue angiotensin-converting enzyme inhibitors [57] like lisinopril or ramipril are our age-independent HF drugs of choice, based on the hypothesis that stimulated RAAS can be blocked by both drugs acting synergistically to lower systemic vascular resistance and avoid myocardial fibrosis, the latter together with the mineralocorticoid-receptor blocker spironolactone.

**Fig. 3:**
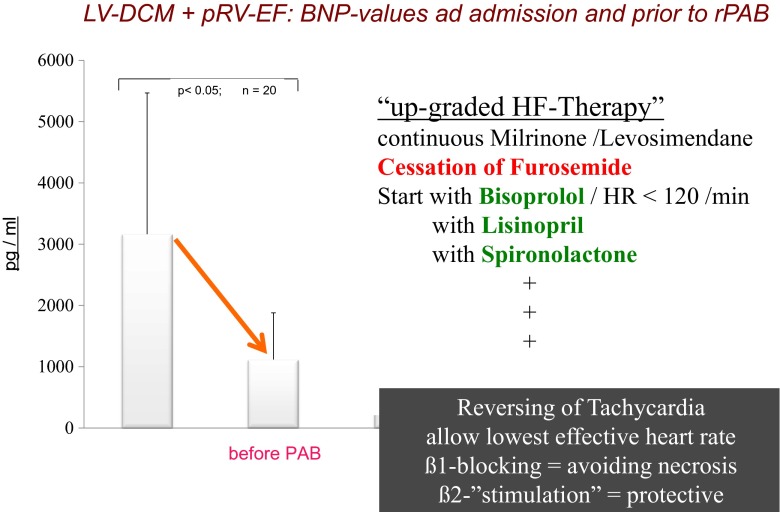
Shown is the mean brain natriuretic peptide (BNP) value of 20 infants and children younger than 3 years with left ventricular dilative cardiomyopathy (LV-DCM) and preserved right ventricular ejection fraction (pRV-EF), which were admitted for heart transplantation. The extremely high BNP values at admission decreased significantly [we believe as a consequence of the administered ‘triple therapy’] during the period prior to surgical pulmonary banding (PAB). At admission, all patients had been treated with in some high dosages of furosemide independent of continuously administered inotropic treatment. Furosemide was stopped and bisoprolol (B), lisionopril (L), and spironolactone (S) were started. The goal was to achieve a resting heart rate (HR) of less than 120/min with an adequate systemic blood pressure to sufficient diuresis. The inotropic treatment was continued, but if dobutamine had been part of the pre-admission regiment, it was changed to the inodilator milrinone

In order to improve systemic blood flow, these drugs have not only been recommended in HF patients with stable hemodynamics [25], but also to transit unstable patients to a stable hemodynamic status [43, 50, 53]. More recently, we are treating all LV-DCM patients with the triple drug combination in preparation for reversible pulmonary artery banding [43, 53]. Only patients with volume depletion due to overdiuresis are excluded, but ongoing inotropic support is not a contraindication for additional ß1-adrenoreceptor blockade, which may facilitate successful weaning of inotropes. Clinical parameters and biomarkers monitor treatment success (Fig. 3). Treatment compliance together with very low side effects, can be impressive, as we could observe and document prior to and immediately after reversible pulmonary arterial banding (rPAB) as a novel therapy to treat LV-DCM [43, 53].

Additional questions arise: why is captopril typically recommended as the first choice for HF treatment in infants and enalapril an appropriate choice for those older than the age of 2 years [24]? In infants and young children, bronchoconstriction and bronchiolitis are common and often associated with HF. Thus, it is our view that non-specific BAR blockers with a ß2-blocking effect and less-specific ACE-I with a higher risk of bradykinin-dependent cough and bronchoconstriction should be avoided. High renin and creatinin levels together with hyponatremia are classical signs not only of severe HF but also of inadequate diuretic therapy. To lower the risk of a blood pressure drop after initiating vasodilator therapy, there should not be a dosage reduction or withdrawal of ACE-I therapy, but rather implementation of diuretics [19, 20, 37, 44, 59].

### Hyperdynamic HF related to a ventricular left-to-right shunt

**Fig. 4:**
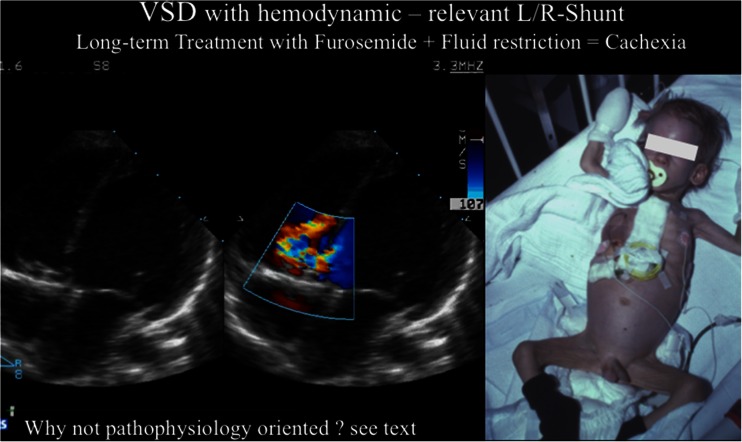
Represents L/R shunt of hemodynamically relevant VSD; infants and young children not early surgically corrected, but mid-term treated by diuretics and fluid restriction develop as pictured severe cachexia because L/R shunt is favored by increased systemic vascular resistance and high oxygen consumption by compensating increased heart and breath rate and in particular of malnutrition

Surgical or transcatheter closure of a hemodynamically relevant ventricle septum defect (VSD) is the treatment of choice. However, there may be several reasons why such a curative approach cannot be performed or need to be delayed. Diuretics, digoxin, and fluid restriction are usually recommended to treat chronic HF based on a left-right shunt, although there is no data that document efficacy [12, 15, 42, 55]. This above management strategy is ill conceived because diuretics, fluid restriction, and digoxin do not achieve the goal of increasing systemic blood flow but instead favor the imbalance of pulmonary and systemic blood flow [12, 13]. Diuretic- and fluid restriction-dependent neuro-humoral stimulation and digoxin-caused vasoconstriction increase the left-right shunt; in mid-term, such treatment might be associated by cardiac cachexia (Fig. 4). And as a reminder, in congenital heart defects with left-right shunt, the systolic function of the left ventricle is mostly preserved [13], whereas diastolic function may be impaired. Older patients with a left-right shunt develop commonly diastolic dysfunction caused by the compensatory mechanism of left ventricular volume depletion due to sympathetic and RAA stimulation. Again, based on our own institutional experience of more than a decade, bisoprolol without blocking the systemic vasodilation favoring ß2-adrenoreceptor, highly specific tissue ACE-I, like lisinopril and spironolactone, if diastolic dysfunction with preserved ejection fraction are present, should be and are used to influence the balance of left-to-right shunt in order to favor systemic blood flow. The improvement of chronic HF symptoms can be easily monitored by the decrease in heart and respiratory rates, signs of less severe pulmonary congestion, and improved food intake in infants. This therapeutic concept avoids cardiac cachexia, which was usually observed in almost all patients in the past and is still observable today.

### HF prophylaxis in hypoplastic left heart syndrome after hybrid stage I

The right ventricle as the systemic heart chamber is usually found in congenital malformations like congenital corrected transposition of the great arteries (ccTGA) and also in the hypoplastic left heart syndrome (HLHS) and in the HLH-complex; in HLHC, the right ventricle works in parallel with an obstructed or borderline left ventricle. There is growing evidence that RV dysfunction develops in many of those patients and accounts for the considerable morbidity and mortality. Therefore, systemic RV function needs close surveillance and sufficient timing of an appropriate intervention to optimize outcome. Almost dogmatically, HF medications, which have been pronounced to be effective for the treatment of a failing left ventricle, are judged to be ineffective for treating a failing systemic right ventricle [24, 25]. However, we wish to point out that the subpulmonary right or left ventricle is metabolically differently active and this may have substantial implications for the pharmacotherapy. Genes encoding drug-metabolizing enzymes, like cytochrome P450 mono-oxygenases, are predominantly expressed in the subpulmonary heart chamber; this might explain the lack of efficacy of drugs like angiotensin-converting enzyme inhibitors and angiotensin receptor blockers on a subpulmonary RV or LV. An atrial switch of the venous connection to the right and left atria reverses the messenger RNA expression profiles. An anatomical left, but subpulmonary positioned, ventricle shows the expression of cytochrome P450 genes normally found in the subpulmonary RV. These facts highlight the importance of the subpulmonary ventricle and pulmonary circulation for the metabolic breakdown of drugs [58]. Thus, for the pharmacological response more important than the morphology of the ventricle appears to be its position. Such data are not available for univentricular hearts, in particular prior to Fontan completion. Considering HLHS, the right ventricle is responsible for the systemic and pulmonary circulation, but following the surgical completion of the Fontan circulation, the distally to the pulmonary circulation positioned RV supports the systemic circulation.

**Fig. 5:**
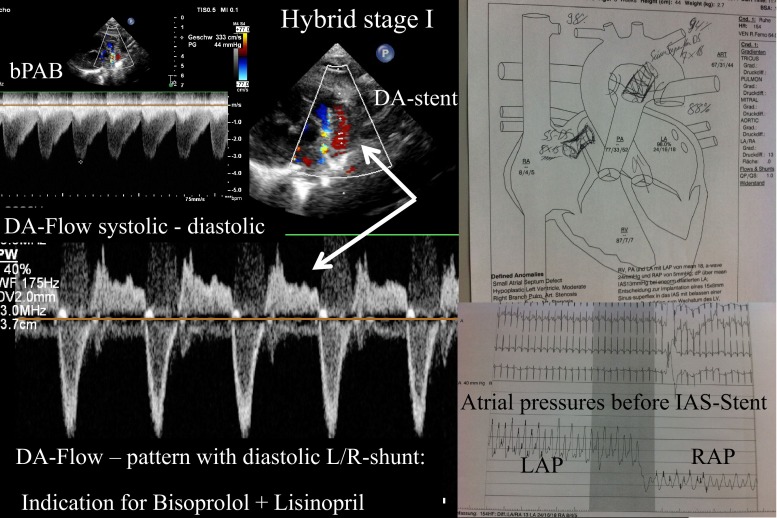
Shows the schematic picture of Hybrid stage I consisting of duct stenting (*DA stent*), bilateral pulmonary banding (*bPAB*), and interatrial septum manipulation by stent placement. Left atrial (*LAP*) decompression to a pressure level of the right atrium (*RAP*) is an important part of a balanced parallel turned pulmonary to systemic circulation. The echocardiography shows an effective bPAB by its typical flow-pattern, but despite an effective bPAB, the systolic right-to-left flow through the duct is accompanied by a diastolic left-to-right reflow; one important indication to reduce the systemic vascular resistance without jeopardizing coronary blood flow. Additionally, bisoprolol reduces heart rate, which improves single ventricle filling; all factors together diminish pulmonary congestion, reduce total and myocardial oxygen consumption, and improve the baby’s functional class

At our institution, neonates born with HLHS and some patients with HLHC are palliated using the Giessen Hybrid approach which consists of bilateral pulmonary banding, duct stenting, and, if necessary, atrial septum manipulation (Fig. 5). This approach has been established more than 17 years ago as an alternative to the Norwood stage I operation [54]. It is our goal to reduce inter-stage morbidity and mortality; one part of this inter-stage strategy is based on the use of dual or triple therapy of bisoprolol with lisinopril and spironolactone. The use of ß1-selective BAR blockers is further supported by recently published data from Miyamoto et al. [34], which stresses the altered BAR signaling in HLHS [34]. During the last 5 years, we have routinely avoided diuretics or digoxin when treating newborns or young infants discharged home after the hybrid procedure. We monitor heart and respiratory rate (Video 1) and, intermittently, systolic and diastolic blood pressure. The mean blood pressure should not be relied on simply because a diminished systemic blood flow might remain undetected. Echocardiographic and serum biomarkers (BNP, ProBNP) ought to be used for additional, intermittent monitoring. The blood pressure amplitude, together with the echocardiographically estimated degree of diastolic left-to-right shunt across the stented duct is important and supports our medical strategy (Fig. 5). The effect of the drug therapy on the systemic vascular resistance can be easily monitored. Low heart rate, prolonged diastolic filling time, and reduction of systemic vascular resistance (without increasing retrograde aortic blood flow and jeopardizing myocardial perfusion pressure) are the therapeutic goals. Excellent parental compliance with a single-dose regiment and the monitoring of body weight prior to hybrid stage II are usually obtained. Whether out-of-proportion myocardial hypertrophy of the systemic right ventricle or interstitial fibrosis can also be reduced by our treatment strategy remains to be examined in future studies.

Video 1With permission of the mother shown here with her premature baby treated with the “Giessen Hybrid” method (see text), the video demonstrates how the parents are advised to monitor her babies that have a high risk of acute clinical deterioration. If the age-dependent respiratory rate at rest is normal the baby is stable; if the respiratory rate becomes fast, for example: double the normal rate, the parents have been advised to contact an experienced doctor or the responsible institution (MOV 2982 kb)

## In conclusion

Our clinical practice and institutional experience in chronic HF treatment in infants and children with left ventricular systolic heart failure, high-output failure due to significant left-right shunting congenital heart diseases, as well as HLHS and HLH-complex after hybrid stage I, are in clear contrast to the official guidelines for chronic HF therapy in infants and children. We acknowledge as a substantial weakness that we are missing randomized study results; however, in the absence of such trials, observational studies should receive credit as a first step. In addition, the here illustrated chronic HF therapy can lay the ground for a randomized multicenter studies designed to analyze the efficacy of other endogenous repair mechanisms supporting therapies as we advocated by pulmonary arterial banding in selected young patients with DCM and preserved right ventricular function.

## Electronic supplementary material

Below is the link to the electronic supplementary material.

## Abbreviations

ACE-I: Angiotensin-converting enzyme inhibitor
ARB: Adrenergic receptor blocker
BAR: ß-Adrenergic receptor
BNP: Brain natriuretic peptide
cHF: Chronic heart failure
CHF: Congestive heart failure
DCM: Dilative cardiomyopathy
HFpEF: Heart failure with preserved ejection fraction
HLHS: Hypoplastic left heart syndrome
HTX: Heart transplantation
LV: Left ventricle
NO: Nitric oxide
NYHA: New York Heart Association
RAA-S: Renin-angiotensin-aldosterone system
RV: Right ventricle
VSD: Ventricle septum defect
VVI: Ventricular-ventricular interaction
